# Glycomics, Glycoproteomics, and Glycogenomics: An Inter-Taxa Evolutionary Perspective

**DOI:** 10.1074/mcp.R120.002263

**Published:** 2021-01-06

**Authors:** Christopher M. West, Daniel Malzl, Alba Hykollari, Iain B.H. Wilson

**Affiliations:** 1 Department of Biochemistry & Molecular Biology, Center for Tropical and Emerging Global Diseases, Complex Carbohydrate Research Center, University of Georgia, Athens, Georgia, USA; 2 Department für Chemie, Universität für Bodenkultur, Wien, Austria; 3 VetCore Facility for Research/Proteomics Unit, Veterinärmedizinische Universität, Vienna, Austria

**Keywords:** Evolution, invertebrate, protist, glycome, glycosyltransferase, EGF, epidermal growth factor, Gal*f*, galactofuranose, GalNAc, *N*-acetylgalactosamine, GlcA, glucuronic acid, GlcNAc, *N*-acetylglucosamine, GPI, glycophosphatidylinositol, GT, glycosyltransferase, HILIC, hydrophilic interaction liquid chromatography, LacdiNAc-, GalNAcβ1,4GlcNAc-, LECA, last eukaryotic common ancestor, MALDI-TOF-MS, matrix-assisted laser desorption ionization mass spectrometry, Neu5Ac, *N*-acetyl-neuraminic acid, Neu5Gc, *N*-glycolyl-neuraminic acid, PC, phosphorylcholine, PE, phosphorylethanolamine, RP-HPLC, reversed-phase high-pressure liquid chromatography, SCF, Skp1/Cul1/F-box class of E3 ubiquitin ligases, TSR, thrombospondin repeat

## Abstract

Glycosylation is a highly diverse set of co- and posttranslational modifications of proteins. For mammalian glycoproteins, glycosylation is often site-, tissue-, and species-specific and diversified by microheterogeneity. Multitudinous biochemical, cellular, physiological, and organismic effects of their glycans have been revealed, either intrinsic to the carrier proteins or mediated by endogenous reader proteins with carbohydrate recognition domains. Furthermore, glycans frequently form the first line of access by or defense from foreign invaders, and new roles for nucleocytoplasmic glycosylation are blossoming. We now know enough to conclude that the same general principles apply in invertebrate animals and unicellular eukaryotes—different branches of which spawned the plants or fungi and animals. The two major driving forces for exploring the glycomes of invertebrates and protists are (i) to understand the biochemical basis of glycan-driven biology in these organisms, especially of pathogens, and (ii) to uncover the evolutionary relationships between glycans, their biosynthetic enzyme genes, and biological functions for new glycobiological insights. With an emphasis on emerging areas of protist glycobiology, here we offer an overview of glycan diversity and evolution, to promote future access to this treasure trove of glycobiological processes.

## Highlights

• Emerging evidence for evolution of eukaryotic protein glycans is summarized
• Six common glycan–protein linkages are predicted in the last eukaryotic common ancestor
• Protists and invertebrates exhibit diverse peripheral glycan modifications
• Cytoplasmic glycosylation frequently occurs in protists

## Graphical Abstract

**Figure:**
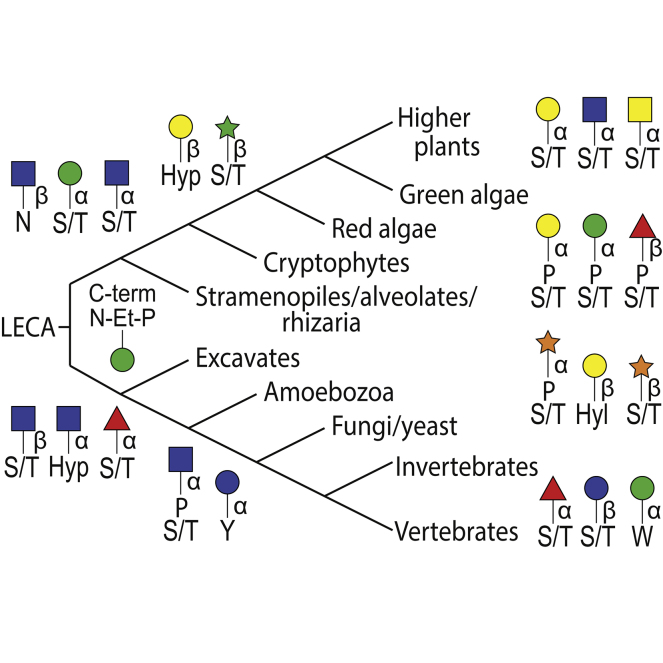

## In Brief

This review article i) assesses the utility of current glycomic, glycoproteomic, and glycogenomic methods to characterize protein glycosylation in less-well-studied eukaryotes; ii) assembles a plausible evolutionary lineage of eukaryotic glycan–protein linkages from the last eukaryotic common ancestor through protists to multicellular plants, invertebrates, and vertebrates; and iii) highlights the diversity of peripheral glycan specializations and modifications with an emphasis on available information from diverse protist kingdoms and invertebrate animals.

**Fig. 1:**
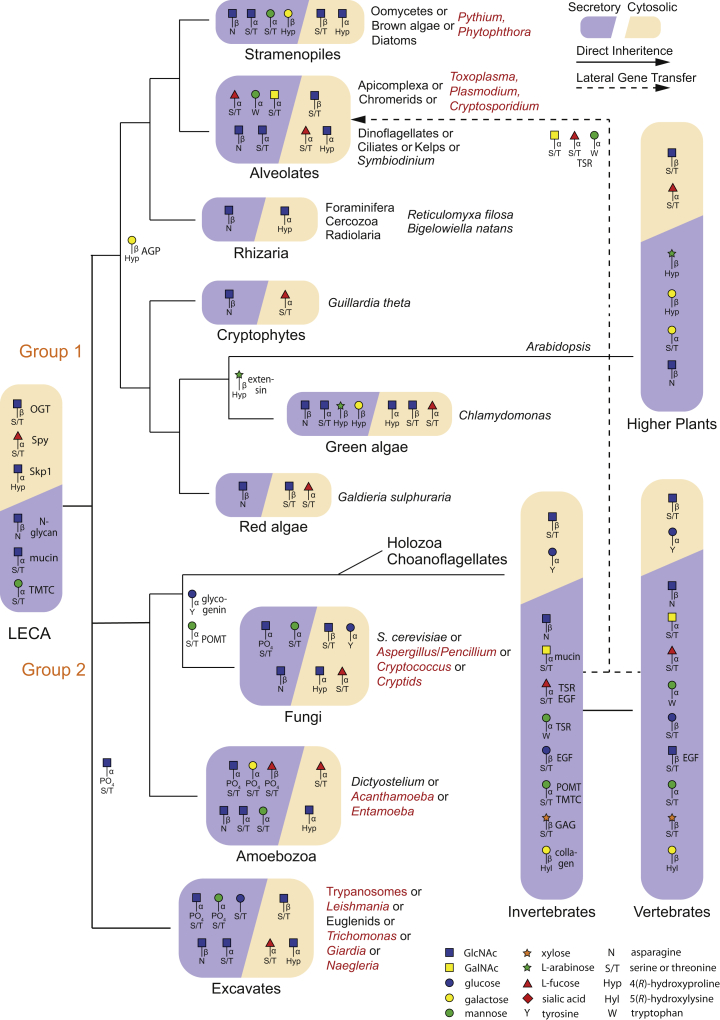
**Evolutionary map of eukaryotic glycans based on the core sugar–amino acid linkage type**. Glycan protein linkages, based on direct evidence or inferred from glycogenomics (see text), are layered on a cladogram from a current model for the phylogeny of eukaryotes (3), which emphasizes the protist subkingdoms and their proposed relation to the last eukaryotic common ancestor (LECA). Major groups where glycomic or bioinformatics information is available are named, with species names in italics. Human or plant pathogens are in red. Linkages found in any one species qualifies for assignment in the group. The dashed line represents likely lateral gene transfer. Poorly studied protists for which there is a lack of experimental data are not shown. Organisms that branched from the lineage that gave rise to the higher plants are referred to as Group 1, whereas those that gave rise to animals are classified as Group 2. Linkages are inferred to occur in the LECA if they are found in both groups of protists, but the absence of a linkage might be a result of incomplete information. The origin of linkages inferred to originate after the LECA is shown at the relevant branch point. Notes adjacent to linkages indicate names by which they are commonly referred. GPI anchors, a specialized glycolipid linked to protein C termini typically *via* a phosphoethanolamine linker to a nonreducing terminal mannose, was likely present in the LECA (not shown). SNFG symbols for sugars are summarized at the bottom. See text for explanations.

Up to half of all eukaryotic proteins are expected to be glycosylated, and while most are secreted or membrane-bound, many are also nucleocytosolic or even mitochondrial. Over the past decades, a range of linkages between sugars and proteins have been discovered. The initial transfer of sugar is determined by primary, secondary, or even sometimes tertiary determinants in the polypeptide structure as well as by the “glycogenomic” capacity of an organism. The glycomes of unicellular, fungal, plant, and animal species diverge highly, although many common elements are found. Several classes of protein glycosylation, defined by the linkage of the reducing terminus to the amino acid, can be traced throughout the protist kingdom and therefore inferred to occur in the last eukaryotic common ancestor (LECA). Of these, N-glycans, GPI anchors, O-GlcNAc and O-Fuc, were retained by higher plants, and N-glycans, GPI-anchors, mucin-type O-glycans, and O-GlcNAc were retained in metazoa. Others had their origins within a protist clade, some of which persisted into plants or animals; others, such as glycosaminoglycans linked to Xyl, appear to be a metazoan “invention.” Here we probe into current knowledge of the evolution of these glycans, except for glycosaminoglycans and GPI-anchors, which have been examined elsewhere (1, 2); in Figure 1 we map different linkages to a phylogenetic tree, whereby ongoing refinements of the protist tree of life are converging on a cladogram whose two major branches provided the plants and animals (3).

## Nature Shuffles the Pack

**Fig. 2:**
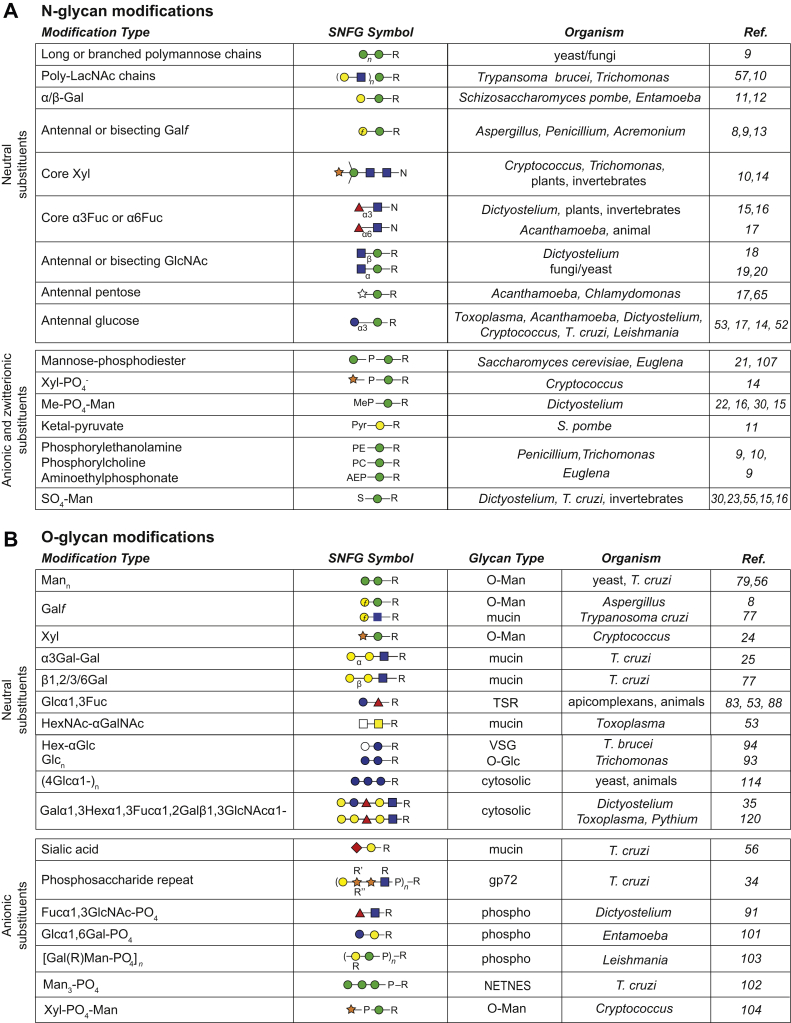
**Peripheral linkages described in protist, fungal and invertebrate glycans**. Examples of peripheral saccharides and their nonsaccharide modifications of protist, fungal, and invertebrate glycans are summarized, together with organisms where found and relevant references (9, 10, 11, 12, 13, 14, 15, 16, 17, 18, 19, 20, 21, 22, 23, 24, 25). *A*, N-glycans. *B*, O-glycans. See Figure 1 for the symbol key and the text for explanations. Empty symbols indicate undetermined sugar isomer. N indicates linkage to Asn and R/R′/R′′ the remainder of a glycan structure.

In mammals, there are nine basic monosaccharide building blocks, and many of these “cards” are also constituents of nonmammalian glycans. Generally, invertebrates lack sialic acid, except for minimal presence in arthropods and wider occurrence in echinoderms (4); in protists, its presence on parasite glycans is due to grabbing this sugar from host structures (5). In plants, N-glycans containing core l-fucose and bisecting xylose are very conserved, while their O-glycans and cell wall polysaccharides are highly diverse (6). Other than Gal*f* in some species (7, 8), there are few reports on “non-canonical” monosaccharides in the protein-linked glycans of invertebrates, fungi, or protists—but nonsugar substituents add to the variety, as summarized in Figure 2 (9, 10, 11, 12, 13, 14, 15, 16, 17, 18, 19, 20, 21, 22, 23, 24, 25). Examples include sulfation, phosphorylation, methylation, pyruvylation, or zwitterionic moieties (4), many of which are not found in mammalian glycans. There are many holes in our knowledge about protein-linked glycans (we will not even start with glycolipids, lipoglycans, or glycolipid anchors!), but our own and other groups have collectively analyzed glycomes from various protists, fungi, and invertebrates, including model organisms, parasites, hosts for parasites, or species of biotechnological relevance.

## The Numbers Game

Our knowledge of glycomes is only as complete as the methodology to define them. Mass spectrometry is currently the major approach and results in sets of *m*/*z* ratios whose structural basis must be interpreted (26, 27). Unlike normally linear peptide chains, whose components have generally different masses, the building blocks of glycoconjugates often have the same mass. Furthermore, the ability of sugars to form branched structures leads to a high theoretical complexity, although conserved biosynthetic pathways of, *e.g.*, N-glycans mean some aspects of the structures are accepted without question. The result is the presence of isomeric and isobaric glycans that are difficult to distinguish—some combinations have exactly the same mass as the atomic composition is identical, others need a highly accurate instrument to be distinguished; in other cases, correct isotopic peak picking or charge state definition must be carefully done. In an era of increasing use of computational annotations, there is the danger that glycan structures can be completely misassigned. Thus, it is useful to consider which combinations of sugar and nonsugar modifications may yield ambiguous masses.

Years ago, it was difficult to distinguish the occurrence of methylated fucose (+160 Da), hexose (+162 Da), or phosphorylcholine (+165 Da) on a linear MALDI-TOF MS. Thankfully, the isotopic resolution of reflectron instruments enables such combinations to be better resolved, but the pattern of isotopic *m*/*z* values should be critically assessed to see whether two glycans of slightly different mass are present in a given sample, never mind that a hexose can be a mannose, a glucose, a galactose, or a galactofuranose. Only the highest-resolution instruments can differentiate phosphate and sulfate (Δ*m*/*z* respectively of 79.9663 and 79.9569 Da), and also methylated hexose and GlcA are very similar (Δ*m*/*z* 176.0685 *versus* 176.0321 Da).

When considering combinations of two building blocks or sialic acids, then the numbers game becomes more complicated: a difference of 291 or 307 Da is indicative of either Neu5Ac or Neu5Gc. However, 292 or 308 Da can be, respectively, due to two fucoses or a fucose and a hexose (more bizarre combinations are also possible). Even the “humble” addition of 324 Da may not be due to two hexoses (2 × 162 Da), but to the presence of an *N*-acetylglucosamine and a methyl-2-aminoethylphosphonate (*i.e.*, 203 + 121 Da). Occasionally unhelpful is the difference in *m*/*z* of sodium or potassium adducts, as 16 Da is also the Δ*m*/*z* for deoxyhexose and hexose.

Naturally, at higher masses, more combinations become possible. Consider Hex_5_HexNAc_4_Fuc_1_ as found in a typical mammalian biantennary N-glycan with two terminal β1,4-galactose and one core α1,6-fucose residues. The corresponding mass, 1786 Da, is identical to a biantennary glycan with terminal β1,3-galactose residues or a hybrid glycan with one LacdiNAc-modified antenna. It can also correspond to a Man_5_GlcNAc_2_ modified with a bisecting and intersecting GlcNAc and a core α1,3-fucose if isolated from *Dictyostelium*. We have previously described other examples of unusual isobaric glycans containing methyl-2-aminoethylphosphonate, methylated hexose, or β-mannose on the reducing GlcNAc (28).

Some of these structures can be distinguished by analysis in both positive and negative modes or employment of chemical or enzymatic treatments. For instance, a glycan with GlcA is not only sensitive to some glucuronidases, but is often detectable in both positive and negative ion modes as is a phosphorylated glycan. Sulfated glycans are often only detected as their molecular ions in negative mode, but tend to suffer from source loss especially in positive ion mode (4). Glycans with a phosphate, phosphonate, phosphodiester, α1,2/3/4-linked fucose, or Gal*f* will lose these units to a greater or lesser extent upon treatment with hydrofluoric acid, which will also lactonize α2,3-linked sialic acid (4, 29). Fractionation of native glycans by porous graphitized carbon (PGC) or HILIC and/or fluorescent labeling combined with RP- or NP (normal phase)-HPLC prior to MS enables enrichment of less abundant structures or separation of many isobaric/isomeric forms (28, 30). Similarly, RP-HPLC separation of permethylated glycans is useful for analyzing isomers (31), but permethylation must involve sulfation-friendly purification methods (32) and may result in loss of zwitterionic glycans. Linkage information *via* cross-ring fragmentation (33), GC-MS, or NMR data also aids structural definition. Certainly, even a composition based on high-resolution mass spectrometry should be backed up by orthogonal proofs (34, 35), especially if novel.

## N-Glycosylation

The GlcNAcβ1-Asn linkage is conserved throughout eukaryotes; the relevant rER oligosaccharyltransferases (OSTs) have their origins in prokaryotes and archaea, though different sugars are transferred. The canonical eukaryotic Glc_3_Man_9_GlcNAc_2_- donor structure appears to have been present in the LECA, but truncated versions are found in various protist taxa, evidently the result of gene loss (36). Thereby, a reduction in the number of mannose residues, and the addition of novel mannose residues, influences the variety of α-mannosidase-processed derivatives that appear. In some branches, terminal glucosylation associated with chaperone-assisted folding is diminished or absent (37).

It is now clear that “lower” animals are capable of synthesizing rather complex N-glycan structures (38). Fucosylation, sulfation, glucuronylation, methylation, or phosphodiesters are rather widespread as antennal modifications, but also galactose or GalNAc can be present. Thereby, mollusks often have methylated glycans, but species-specific differences include antennal methylated blood group A motifs or disubstituted fucose residues (39, 40, 41). In nematodes, cestodes, and lepidoptera, phosphorylcholine occurs, but in the honeybee, phosphoethanolamine is found (38, 42). On the other hand, schistosomes rather “play” with fucose (43). Some marine organisms are rich in sulfated glycans, whether they be mollusks or echinoderms (39, 44). Sulfation is found in insects too, but the positions vary, and we have found sulfate on mannose, fucose, GlcNAc, and galactose residues in different invertebrates (39, 44, 45). GlcA may be the nearest equivalent to sialic acid in many insects (42) and is also found on the N-glycan antennae of (at least) one nematode (46). Sialic acid is undoubtedly present on N-glycans of *Drosophila* (47) and echinoderms (29).

The core regions of N-glycans can be highly decorated, which means that there is more than just core α1,6-fucosylation, but also galactosylation or sulfation of the α1,6-fucose, core α1,3-fucosylation of either GlcNAc of the chitobiose unit or β-mannosylation of the reducing terminus (38, 42). Indeed, difucosylation of this core GlcNAc is a rather common feature of invertebrates (38). Additionally, the core mannose in schistosomes and some molluscs can carry a xylose as in plants (48). *Caenorhabditis elegans* intriguingly possesses the most extremely modified N-glycan cores, of those found to date, with multiple fucose and galactose residues (49).

In the protist realm, the diversity is no less bewildering, with smaller versions all the way down to the GlcNAc_2_ core being transferred from the lipid-linked donor in various protist taxa (50, 51). Glc residues that are typically removed after transfer to protein in animals are sometimes retained (52, 53), suggesting the absence of a role in quality control (37). Although the metazoan enzymes for processing hybrid and complex forms appear absent, GlcNAc-based antennae reminiscent of complex forms are found in various protists and fungi (Fig. 2), and more examples are likely (*e.g.*, (54, 55, 56)). Atypical GlcNAcTs have been identified in *Trypanosoma brucei* (57, 58), a finding that shows that GT families can include “reassigned” regio-specificities, which complicates glycophylogenetic comparisons based on genome analyses. Further antennal modifications include long and even branched polymannose chains, atypically linked neutral sugars, poly-LacNAc chains, Gal*p,* Gal*f*, Xyl, and a variety of anionic substituents including methylphosphate, sulfate, and pyruvate (Fig. 2*A*). On the other hand, in some protist species, there are modifications of the core with xylose and fucose as in other taxa. The detection of many of these structures historically involved release by endoglycosidases, radiolabeling, exoglycosidase digestion, co-chromatography, and when possible, NMR; the advent of mass spectrometry coupled with prior knowledge of structures and the specificities of the enzymes that assemble them has allowed us to go a very long way to define structures, but often correlation with the presence or involvement of glycosylation genes is necessary for explication, as illustrated by a recent study in *Toxoplasma gondii* (53). However, the diversity of structures that exist is still underestimated, because of limitations in enzymatic release or their ionization and fragmentation in the mass spectrometer. Furthermore, when applied to whole-cell homogenates, these methods tend to favor the detection of the most abundant glycans leaving open the question of rarer protein-specific variants.

The orthogonal approach of analyzing peptides by mass spectrometry can help address these limitations. Typical proteomics workflows, involving proteases such as trypsin, pepsin, Pronase, or proteinase K and nano-RP-HPLC-MS, will measure the mass of N-glycosylated peptides and present a preliminary structural assessment based on prior glycomic knowledge. This of course also provides site-specific information, and the near universal use of the N-sequon (NxS/T/sometimes C, x not P) is a valuable predictor. Notably, *T. brucei* has two OSTs with distinct preferences for different lipid-linked donors and flanking residues preceding the N-sequon (59). In practice, suppression of glycopeptides during ionization and low abundance of isoforms owing to glycan microheterogeneity usually mitigate against success. These limitations are partially overcome by scanning for sugar fragment ions (*e.g.*, characteristic oxonium ions) or pre-enrichment of glycopeptides by HILIC or lectins; however, caution is needed as these introduce bias due to selectivity for glycan types. Prepurification of a target protein if sufficient natural material is available and top-down approaches enable a more comprehensive profiling of glycan types, attachment sites, and site-specific heterogeneity.

Glycoproteomic studies of nonmammalian eukaryotes are certainly underdeveloped, with the most work directed toward plants such as *Arabidopsis* and model organisms such as *C. elegans* (60, 61), but also some data on invertebrate and protist parasites. Using lectin pre-enrichment of glycoproteins or glycopeptides, 141 N-glycan sites were characterized in the parasite *T. brucei* (59), more than a thousand N-glycan sites were more recently identified on many hundreds of proteins in *Trypanosoma cruzi* (62), and over 100 were described in an older study in *T. gondii* (63). In a single *Dictyostelium discoideum* cell surface glycoprotein, 14 N-glycan types detected by MALDI-TOF-MS were mapped to 15 of 18 predicted N-sequons in HILIC- or ConA-enriched peptides, and notably, substantial but differential microheterogeneity was detected at all 15 sites (64). Lectin enrichment was also employed to explore the N-glycoproteome of an algal species (65). Because of the sheer magnitude of the glycoproteomic inventory, it is often prudent to pursue glycan features based on a biochemical or cellular function of interest. This can be explored in various ways, including mutating the biosynthetic enzyme gene or the attachment site, use of glycan hapten inhibitors, selective enzymatic or chemical perturbation of target glycans, selective steric blockade of glycans with lectins or carbohydrate binding proteins, etc. Orthogonal approaches are typically required to circumvent non-specific effects of these methods toward the glycan of interest. However, as the N-glycans of protists are generally sufficiently similar to those of animals, yeast, or plants, lessons learned from multicellular organisms can be applied. With improvements in the ability to predict GTs and processing enzymes, genomic predictions can also be brought to bear on the challenge (37, 53, 65, 66, 67, 68), with the caveat that many genes especially in protists have unknown functions.

## O-Glycosylation

As in mammals, O-glycans occur in many different “flavours” in invertebrates and protists. Typical are the mucin-type O-glycans with αHexNAc attached to serine or threonine. These cell surface and extracellular structures often conserve negative charges with different sugars or substituents. More specialized are the short, usually neutral glycans of folded epidermal growth factor (EGF) and thrombospondin repeat (TSR) domains, whereas very widespread are monosaccharide modifications of nucleocytosolic proteins. As O-glycans are less easy to release and recover than N-glycans, we have far less knowledge about their structural diversity, but the origin of some can be traced back to extant members of the holozoan and choanoflagellate predecessors of invertebrates and others through various protist lineages all the way back to the LECA (69). The sugar–amino acid linkages are the most primal and can be mapped onto the evolutionary tree (Fig. 1), whereas the peripheral sugars in protists show such diversity that we simply summarize their nature (Fig. 2*B*).

## Mucin-Type O-Glycosylation

In animals, including invertebrates, mucin-type O-glycosylation is initiated by a family of up to 20 Golgi-associated CAZy GT27 polypeptide (pp) αGalNAcTs (70), often resulting in high-density arrays on mucin domains rich in Ser and Thr residues. In protists, these are typically initiated with GlcNAc rather than GalNAc, a difference that may correlate with the evolutionary emergence of a UDP-GalNAc epimerase activity (71). Based on studies in *Dictyostelium* and *T. cruzi*, a new family of related pp-αGlcNAcTs (CAZy GT60) was recognized that varies mainly in the absence of the characteristic C-terminal ricin-like targeting domain found in almost all metazoan pp αGalNAcTs (72, 73, 74). Up to four pp-αGlcNAcT genes are found in the genomes of both Group 1 (diatom, oomycete and algae) and Group 2 protists (amoebozoa, *T. cruzi*), indicating its occurrence in the LECA and suggesting target specialization as occurs in the metazoan pp αGalNAcT family. An exception occurs in apicomplexans, where up to five pp-αGalNAcTs are found (75, 76); owing to their absence in other protist genomes (74), these are likely the result of lateral gene transfer (74) perhaps facilitated by residence in animal host cells. Consistent with this hypothesis, the *Toxoplasma* O-glycans are short, consisting of either a single αGalNAc (known as the Tn antigen in humans) or a HexNAc-αGalNAc (53).

Mucin-type protist O-glycans have been particularly well characterized in *T. cruzi* where they are found in extensive arrays on cell surface GPI-anchored mucins. They are extended by α- or β-linked Gal*p* and Gal*f* residues in linear and branched chains, with a prevalence of 4-, 2-, and 6-linkages (77), as compared with the β3- and β6-linkages typical of animal O-glycans. The βGal*p* termini are frequently capped with α3-linked Gal, or sialic acid derived from host glycans by parasite transialidases (5), creating O-glycans with general resemblance to host glycans but distinct in detail. Dozens of isoforms were defined by NMR (77), with recent MS profiling (31, 62) confirming the high variation among *T. cruzi* strains. Interestingly, related GT genes are present in other trypanosomatids but the O-glycans have not been detected biochemically (73), indicating a limitation of global glycomic approaches. The amoebozoan *Dictyostelium* also extensively modifies its mucin-type domains with O-αGlcNAc, contributing to the *modB*-epitope (31), with evidence that some are capped with αFuc (78). The mucin-type O-glycans of protists in Group 1 remain to be characterized.

## O-Man

Another sugar that initiates O-glycans on Ser and Thr residues is α-mannose. In fungi, this linkage replaces the common mucin-type HexNAc-linkage (79) and is linearly extended by further mannoses or Gal*f*. Related protein O-mannosyltransferases (POMTs) are present in most animals, where the Man can be extended by GlcNAc, Gal, and sialic acid in vertebrate mucin domains. A specialized phosphorylated subset carries matriglycan, which modifies α-dystroglycan in mammalian muscle and brain (80). A glycoproteomics study discovered nonextended O-Man on mammalian adhesion proteins, transferred by a separate family of CAZy GT105 TMTC mannosyltransferases in the rER (81). Bioinformatics searches suggest that the POMTs originated in fungi, though a distant homolog in bacteria suggests a more primitive origin (82), while TMTC-like genes are rather widely distributed in both Group 1 and 2 protists and thus probably in the LECA, and potentially explain the origin of a dimannose species detected in *T. cruzi* (56).

## O-Fuc, O-Glc, O-Xyl, O-GlcNAc, and C-Man

These are short, often neutral, one-to-four-sugar glycans on EGF and TSR domains of animal proteins. O-fucose was originally detected by mass spectrometry of human urine samples and later on EGF and TSR repeats of Notch and thrombospondin (83). O-fucosylation is initiated on folded EGF domains in the rER by POFUT1 (CAZy GT68) and can be extended out to a tetrasaccharide capped by sialic acid. EGF repeats are also modified by a small family of GlcTs (84) that can also use UDP-Xyl as a donor (85), and the Glc can be extended by xylose in animals (86). Extracellular β-linked O-GlcNAc is another modification of animal EGF domains, but does not appear to be extended. It is assembled by the CAZy GT61 family member EOGT (87) that is unrelated to αGlcNAcT and βGlcNAcT enzymes described above and below. Some related enzyme sequences in protists and plants have distinct activities.

The O-Fuc found on folded TSRs is applied by POFUT2 and can be capped by a β3-linked Glc assembled by a CAZy GT31-family GT (53). This disaccharide and both GTs are also found in *T. gondii* and *Plasmodium* (agent for malaria) (88), but the genes are otherwise not found in protists. C-Man is a monosaccharide modification of Trp residues typically associated with TSR repeats in metazoan proteins (89). The C-ManT resides in the rER and utilizes Dol-P-Man as the donor and is also present in *Toxoplasma* and *Plasmodium*. C-ManT-like sequences, which belong to CAZy GT98 and the larger GT-C superfamily of Dol-P-sugar-dependent GTs (90), are seen in the broader group of alveolates, and in metazoan progenitors (choanoflagellates and holozoans), but not elsewhere. Thus, C-ManT, POFUT2, and the β3GlcT were potentially, like the pp-αGalNAcTs, acquired by lateral gene transfer (Fig. 1).

O-Fuc has also been reported on secreted proteins in *Dictyostelium* (78, 91, 92). O-Glc has been found in *Trichomonas vaginalis*, where it can be extended with predominantly Glc (93), whereas Glcα-Ser, sometimes extended with additional hexoses (94), was recently discovered on *T. brucei* VSG surface proteins by crystallography and confirmed by mass spectrometry. The enzymatic basis for these protist versions is unknown.

## Hydroxyproline-Linked

Hydroxyproline (Hyp)-linked glycans are the predominant class of O-linked glycans on cell wall proteins of plants and green algae, which lack the O-αHexNAc and O-αMan types common in animals and other protists. Hyp-linked O-glycans depend on prior posttranslational conversion of prolyl residues to 4(*trans*)-hydroxyproline (95). Two major types are found on extensins: a monosaccharide modification consisting of αGal and oligoarabinosyl modifications on adjacent Hyp residues initiated with β-L-Ara. In addition, Gal-Ser and pentose-Hyp linkages have also been detected by mass spectrometry (96). O-glycosylation of arabinogalactan-proteins is much more complex, being initiated by a βGalT and consisting generally of a β3-galactan backbone to which are appended β6-linked Gal chains modified by a variety of monosaccharides including GlcA, resulting in immense structures that dwarf the carrier protein (95). The initiating GTs and some of the extending GTs have been recently identified, and their sequences will help determine the evolutionary origins of these modifications.

Hyp-linked glycans are also found in the green alga *Chlamydomonas* as short linear oligosaccharides of α-l-Ara*f*, as determined by NMR and genomics analyses (97, 98). Sequences related to the CAZy GT31 Hyp:βGalT are found in other Group 1 protists including a Cryptophyte and Stramenopiles, supporting evidence for arabinogalactans in *Fucus* (99). Similarly, the initiating Hyp:βAraT and Ser:αGalT are related to CAZy GT8 and GT96 family members in other protists. Because Hyp-linked O-glycans (as well as hydroxylysine (Hyl) O-glycans as in collagen) resist release by β-elimination (100), searches for their phylogenetic distribution will likely require glycoproteomic aided by glycogenomic approaches.

## Sugar Phospho-Linkages

Phosphodiester-linked glycans originate by transfer of a sugar phosphate, rather than a sugar alone, from sugar nucleotides to Ser or Thr, and are subject to extension in the Golgi by various GTs or other phosphoglycosyltransferases. They are amenable to release as anionic glycans using nonreductive β-elimination and analysis by negative ion mode mass spectrometry, though sugars with 2-OH α-*manno* configuration are susceptible to release as free sugars, or by aqueous HF, which preferentially cleaves the sugar–phosphate linkage (28). The simple structures GlcNAcα1-PO_4_-, Fucα1,3GlcNAc-PO_4_-, and Fucβ1-PO_4_- have been identified in the amoebozoan *Dictyostelium* (91, 92). Another amoebozoan, *Entamoeba histolytica*, assembles a phosphodiester-linked Glcα1,6Galα1-PO_4_- onto Ser/Thr residues and is elongated by additional α6-linked Glc residues (101). A phosphodiester-linked tri-α-mannoside has been described in *T. cruzi* on NETNES (102).

Possibly the most complex protein-linked oligosaccharide has been described on *T. cruzi* Gp72, which is a repeating phosphodiester-linked tridecasaccharide containing l-Rha*p*, l-Fuc*p*, d-Gal*p*, d-Gal*f*, d-Xyl*p*, and d-GlcNAc*p* linked to Thr/Ser *via* αGlcNAc-PO_4_ (34). In another trypanosomatid, *Leishmania*, O-glycosylation occurs in the form of long, diverse, and complex (linear or branched) proteophosphoglycans anchored *via* αMan-PO_4_-Ser/Thr that form extensive networks contributing to a type of biofilm (103). The challenge of detecting and characterizing such complex structures invites speculation that they are more common than assumed. These phosphodiester-linked structures have not been described in metazoa and presumably represent species-specific adaptations that ensure, in the absence of sialylation, a high negative charge density on their cell surfaces. In general, the evolution of phosphosugar structures and the relevant phosphoGTs are highly under-explored.

Phosphodiester-linked sugars also occur as peripheral linkages (Fig. 2). For the pathogenic yeast *Cryptococcus neoformans*, the phosphoGT that assembles a Xyl-PO_4_-Man linkage has been identified (104), while in *Saccharomyces cerevisiae*, Mnn6p is a proven mannosylphosphate transferase toward α2-linked mannose and is related to conventional α-mannosyltransferases (105). In *Dictyostelium*, the enzyme that phosphorylates lysosomal enzymes is orthologous to animal examples (106), and extracts of *Euglena* showed a similar enzymatic activity (107).

## O-βGlcNAc, O-αFuc, and O-αGlc (Nucleocytoplasmic)

O-βGlcNAc is a monosaccharide modification of Ser or Thr residues of thousands of proteins that reside in the cytoplasm and nucleus. Originally discovered in mammals by labeling with β4-galactosyltransferase in conjunction with β-elimination, its distribution can now be mapped directly using ETD mass spectrometry to preserve its sensitive linkage during fragmentation (108). O-βGlcNAc addition is mediated by the action of a CAZy GT41 family member referred to as OGT or Secret Agent (SEC). O-βGlcNAc has a variety of functions in metabolic sensing and regulation, and key to its actions in animals is its removal and therefore cycling by the action of a single O-GlcNAcase known as OGA. Outside of animals, O-βGlcNAc has been most studied in higher plants where genetic studies have elucidated complex roles in modulating plant hormone signaling (109), but the lack of evidence for an OGA suggests that fundamental mechanisms may diverge. OGT-like sequences apparently originated in prokaryotes and are distributed in many Group 1 and 2 protists (110). Its absence in some major groups is most consistent with gene loss, and it might be compensated by other monosaccharide modifications. Given its evident occurrence in the LECA, O-βGlcNAc action may be widespread in protists.

An ancient gene duplication, probably in prokaryotes (110), resulted in a closely related enzyme now known to generate O-αFuc on Ser and Thr. This modification was first characterized on AAL-enriched nucleocytoplasmic proteins of *T. gondii* using mass spectrometry (111). The O-αFuc modification has been detected on dozens of *T. gondii* proteins, multiple *Dictyostelium* proteins, and two FG-repeat nuclear pore proteins of *Cryptosporidium* ( (112), unpublished). Known as Spy or OFT, the enzymatic activity was first described in *Arabidopsis* (113) where prior genetic analyses had demonstrated roles in modulating hormone signaling (110). Subsequent studies demonstrated OFT activity in *T. gondii* and a role in promoting growth (53). Plants express both OGT and OFT, but evidence for OFT in animals or their coexistence in protists has yet to be described.

Finally, yeast and animal glycogen often exists as an O-glycan linked at its reducing terminus to a critical Tyr residue within glycogenin, an autocatalytic α-glucosyltransferase that primes glycogen synthesis in the cytoplasm (114). As for Hyp- and Hyl-linked O-glycans described above, Tyr-linked glycans are not released by β-elimination. Currently thought to occur only in metazoa and fungi, a close homolog and potential evolutionary progenitor of glycogenin contributes to Skp1 glycosylation in Group 1 protists (see below). Distinct nucleocytoplasmic glycans might also exist elsewhere, as suggested by a report of O-Man mono- and oligosaccharides in yeast (115).

## Hyp (Nucleocytoplasmic)

Numerous protists express a complex O-glycan linked to the side chain of 4(*trans*)-hydroxyproline (Hyp) of a nucleocytoplasmic protein, Skp1, which is an essential subunit of the eukaryote-wide SCF class of E3 polyubiquitin ligases, but is glycosylated only in Group 1 and 2 protists. Skp1 glycosylation appears to promote assembly of SCF subcomplexes (116) by a local conformational control mechanism (35, 117, 118). Where the structure has been studied, the glycan consists of a linear pentasaccharide composed of GlcNAc, βGal, αFuc, αGlc (in some species), and αGal. The glycan was initially detected in *Dictyostelium* by metabolic labeling with [^3^H]Fuc, but structural studies required traditional glycoproteomic approaches as well as analysis of the glycopeptide after permethylation (35), a method not generally recognized for its applicability to glycopeptides (119). Exoglycosidase treatments, characterization of the specificity of the GTs on model substrates, and ultimately NMR were required to establish the structures (35, 120). Like many sugar nucleotide-dependent GTs that target proteins in prokaryotes, the Skp1 GTs exist as noncomplexed soluble enzymes in the cytosol and are evolutionarily related to familiar animal Golgi GTs. While the initiating GT for *Dictyostelium* Skp1 is a pp-αGlcNAcT related to its Golgi counterpart that initiates mucin-type O-glycosylation (72), the next two GT activities of *Dictyostelium*, *Toxoplasma*, and *Pythium* (a β3GalT and an α2FucT) (121) are in the same bifunctional protein and belong respectively to the CAZy GT2 and GT74 families whose members are prevalent in prokaryotes (74). In *Toxoplasma*, addition of the fourth sugar is catalyzed by a CAZy GT31 family α3GlcT (Glt1) that is related to Golgi αManTs that elongate mannans in fungi and yeasts (122), and the final sugar is applied by an α3GalT (Gat1) whose CAZy GT8 relatives are often found in the cytoplasm. Gat1 is closely related to the aforementioned glycogenin (120).

In *Dictyostelium*, Glt1 and Gat1 are replaced by an unrelated CAZy GT77 α3GalT (AgtA) that operates twice on the reducing end terminus to also generate a pentasaccharide (123). AgtA is related to an uncharacterized Golgi enzyme in *T. cruzi* and pectin synthesizing enzymes in plants, but in *Dictyostelium*, a C-terminal WD40-repeat β-propeller domain both modulates Skp1 activity independent of its glycosylation status and facilitates addition of the second sugar (123). Interestingly, odd–even enzyme pairs of the 6-enzyme Skp1 modification pathway are frequently expressed as fusion proteins in different protist taxa (121, 124), possibly supporting processive processing. The Skp1 enzymes were evidently present in the LECA and might have contributed to the newly emerging secretory pathway compartments *via* the simple exigency of gene duplication and acquisition of N-terminal signal anchor sequences. Indeed, the Skp1 and Golgi pp-αGlcNAcTs are each encoded by two-exon genes with an intron separating a short N-terminal sequence from the catalytic domain (72, 125).

The unusual linkage of the glycan to a Hyp is related to its function in O_2_-sensing in *Dictyostelium* and *Toxoplasma* (126). Glycosylation is contingent upon the presence of sufficient ambient O_2_ as a substrate for a dedicated prolyl 4-hydroxylase (PhyA) related to the O_2_-sensing PHD2 that regulates HIFα in animals. The evidence suggests that O_2_-sensing had its origins in protists before the enzyme redirected its target from an E3 Ub ligase to the HIFα transcriptional cofactor, whose hydroxylation renders it a target for an evolutionarily related E3 Ub ligase. The genes for this pathway are found in many, but not all, aerobic protists including several human and crop pathogens.

## The Bottom Line

A primary motto in nonmammalian glycomics is “expect the unexpected.” Therefore, if you are more used to analyzing mammalian glycans, a database-centered approach to annotation can yield misleading results. Keeping an open mind, one can begin with separating different classes of glycans using different PNGases and/or fractionation into neutral, anionic, or hydrophobic pools, whereas O-glycan release must be done chemically or at the peptide level for linkages to Hyp, Hyl, or Tyr. Chromatography is highly useful for separating isomeric and isobaric structures before mass spectrometry, whereby chemical and exoglycosidase treatments as well as defined standards ease interpretation. Often, orthogonal information from lectin or antibody recognition or dependence on a GT with characterized specificity forms the basis for inferring a structural element. Evolutionary and glycophylogenetic considerations also come into play, based on the presence of linkage types and/or GTs in related, predecessor, or derivative groups, potentially tracing back to the LECA. Despite the availability of all predicted glycogenes for a few protists (53, 66, 67), we are still a long way from being able to infer the biochemical function of many glycogene paralogs and, thereby, the glycome based solely on genetic information. If your “knowledgebase” is firmly established, then your own computer- and/or brain-based database can be a start for comparisons with other organisms or for glycoproteomics. Together with the nascent technology of natural glycan arrays, then we can start to think about the functions for all this glycobiodiversity!

## Conflicts of interest

The authors declare no competing interests.

## References

[1] Hashimoto K., Tokimatsu T., Kawano S., Yoshizawa A.C., Okuda S., Goto S., Kanehisa M. (2009). Comprehensive analysis of glycosyltransferases in eukaryotic genomes for structural and functional characterization of glycans. Carbohydr. Res..

[2] Corfield A.P., Berry M. (2015). Glycan variation and evolution in the eukaryotes. Trends Biochem. Sci..

[3] Burki F., Roger A.J., Brown M.W., Simpson A.G.B. (2020). The new tree of eukaryotes. Trends Ecol. Evol..

[4] Paschinger K., Wilson I.B.H. (2020). Anionic and zwitterionic moieties as widespread glycan modifications in non-vertebrates. Glycoconj. J..

[5] Freire-de-Lima L., Fonseca L.M., Oeltmann T., Mendonça-Previato L., Previato J.O. (2015). The trans-sialidase, the major *Trypanosoma cruzi* virulence factor: Three decades of studies. Glycobiology.

[6] Nguema-Ona E., Vicré-Gibouin M., Gotté M., Plancot B., Lerouge P., Bardor M., Driouich A. (2014). Cell wall O-glycoproteins and N-glycoproteins: Aspects of biosynthesis and function. Front. Plant Sci..

[7] Beverley S.M., Owens K.L., Showalter M., Griffith C.L., Doering T.L., Jones V.C., McNeil M.R. (2005). Eukaryotic UDP-galactopyranose mutase (GLF gene) in microbial and metazoal pathogens. Eukaryot. Cell.

[8] Tefsen B., Ram A.F.J., van Die I., Routier F.J. (2012). Galactofuranose in eukaryotes: Aspects of biosynthesis and functional impact. Glycobiology.

[9] Hykollari A., Eckmair B., Voglmeir J., Jin C., Yan S., Vanbeselaere J., Razzazi-Fazeli E., Wilson I.B., Paschinger K. (2016). More than just oligomannose: An N-glycomic comparison of *Penicillium* species. Mol. Cell. Proteomics.

[10] Paschinger K., Hykollari A., Razzazi-Fazeli E., Greenwell P., Leitsch D., Walochnik J., Wilson I.B.H. (2012). The N-glycans of *Trichomonas vaginalis* contain variable core and antennal modifications. Glycobiology.

[11] Gemmill T.R., Trimble R.B. (1996). *Schizosaccharomyces pombe* produces novel pyruvate-containing N-linked oligosaccharides. J. Biol. Chem..

[12] Magnelli P., Cipollo J.F., Ratner D.M., Cui J., Kelleher D., Gilmore R., Costello C.E., Robbins P.W., Samuelson J. (2008). Unique Asn-linked oligosaccharides of the human pathogen *Entamoeba histolytica*. J. Biol. Chem..

[13] Ohta M., Emi S., Iwamoto H., Hirose J., Hiromi K., Itoh H., Shin T., Murao S., Matsuura F. (1996). Novel β-D-galactofuranose-containing high-mannose type oligosaccharides in ascorbate oxidase from *Acremonium* sp HI-25. Biosci. Biotechnol. Biochem..

[14] Park J.N., Lee D.J., Kwon O., Oh D.B., Bahn Y.S., Kang H.A. (2012). Unraveling unique structure and biosynthesis pathway of N-linked glycans in human fungal pathogen *Cryptococcus neoformans* by glycomics analysis. J. Biol. Chem..

[15] Hykollari A., Balog C.I., Rendić D., Braulke T., Wilson I.B.H., Paschinger K. (2013). Mass spectrometric analysis of neutral and anionic N-glycans from a *Dictyostelium discoideum* model for human congenital disorder of glycosylation CDG IL. J. Proteome Res..

[16] Feasley C.L., van der Wel H., West C.M. (2015). Evolutionary diversity of social amoebae N-glycomes may support interspecific autonomy. Glycoconj. J..

[17] Schiller B., Makrypidi G., Razzazi-Fazeli E., Paschinger K., Walochnik J., Wilson I.B.H. (2012). Exploring the unique N-glycome of the opportunistic human pathogen *Acanthamoeba*. J. Biol. Chem..

[18] Couso R., van Halbeek H., Reinhold V., Kornfeld S. (1987). The high mannose oligosaccharides of *Dictyostelium discoideum* glycoproteins contain a novel intersecting N-acetylglucosamine residue. J. Biol. Chem..

[19] Buser R., Lazar Z., Käser S., Künzler M., Aebi M. (2010). Identification, characterization, and biosynthesis of a novel N-glycan modification in the fruiting body of the basidiomycete *Coprinopsis cinerea*. J. Biol. Chem..

[20] Smith W.L., Nakajima T., Ballou C.E. (1975). Biosynthesis of yeast mannan. Isolation of *Kluyveromyces lactis* mannan mutants and a study of the incorporation of N-acetyl-D-glucosamine into the polysaccharide side chains. J. Biol. Chem..

[21] Hernandez L.M., Ballou L., Alvarado E., Tsai P.K., Ballou C.E. (1989). Structure of the phosphorylated N-linked oligosaccharides from the mnn9 and mnn10 mutants of *Saccharomyces cerevisiae*. J. Biol. Chem..

[22] Gabel C.A., Costello C.E., Reinhold V.N., Kurz L., Kornfeld S. (1984). Identification of methylphosphomannosyl residues as components of the high mannose oligosaccharides of *Dictyostelium discoideum* glycoproteins. J. Biol. Chem..

[23] Freeze H.H., Wolgast D. (1996). Structural analysis of N-linked oligosaccharides from glycoproteins secreted by *Dictyostelium discoideum*. Identification of mannose 6-sulfate. J. Biol. Chem..

[24] Lee D.J., Bahn Y.S., Kim H.J., Chung S.Y., Kang H.A. (2015). Unraveling the novel structure and biosynthetic pathway of O-linked glycans in the Golgi apparatus of the human pathogenic yeast *Cryptococcus neoformans*. J. Biol. Chem..

[25] Soares R.P., Torrecilhas A.C., Assis R.R., Rocha M.N., Moura e Castro F.A., Freitas G.F., Murta S.M., Santos S.L., Marques A.F., Almeida I.C., Romanha A.J. (2012). Intraspecies variation in *Trypanosoma cruzi* GPI-mucins: Biological activities and differential expression of α-galactosyl residues. Am. J. Trop. Med. Hyg..

[26] Feasley C.L., Hykollari A., Paschinger K., Wilson I.B., West C.M. (2013). N-glycomic and N-glycoproteomic studies in the social amoebae. Methods Mol. Biol..

[27] Hykollari A., Malzl D., Wilson I.B.H., Paschinger K. (2019). Protein-specific analysis of invertebrate glycoproteins. Methods Mol. Biol..

[28] Paschinger K., Wilson I.B.H. (2016). Analysis of zwitterionic and anionic N-linked glycans from invertebrates and protists by mass spectrometry. Glycoconj. J..

[29] Eckmair B., Jin C., Karlsson N.G., Abed-Navandi D., Wilson I.B.H., Paschinger K. (2020). Glycosylation at an evolutionary nexus: The brittle star *Ophiactis savignyi* expresses both vertebrate and invertebrate *N*-glycomic features. J. Biol. Chem..

[30] Hykollari A., Malzl D., Yan S., Wilson I.B.H., Paschinger K. (2017). Hydrophilic interaction anion exchange for separation of multiply modified neutral and anionic *Dictyostelium* N-glycans. Electrophoresis.

[31] Sheikh M.O., Gas-Pascual E., Glushka J.N., Bustamante J.M., Wells L., West C.M. (2019). *Trypanosoma cruzi*^13^C-labeled O-glycan standards for mass spectrometry. Glycobiology.

[32] Kurz S., Aoki K., Jin C., Karlsson N.G., Tiemeyer M., Wilson I.B.H., Paschinger K. (2015). Targeted release and fractionation reveal glucuronylated and sulphated N- and O-glycans in larvae of dipteran insects. J. Proteomics.

[33] Ashline D.J., Zhang H., Reinhold V.N. (2017). Isomeric complexity of glycosylation documented by MSn. Anal. Bioanal. Chem..

[34] Allen S., Richardson J.M., Mehlert A., Ferguson M.A.J. (2013). Structure of a complex phosphoglycan epitope from gp72 of *Trypanosoma cruzi*. J. Biol. Chem..

[35] Sheikh M.O., Thieker D., Chalmers G., Schafer C.M., Ishihara M., Azadi P., Woods R.J., Glushka J.N., Bendiak B., Prestegard J.H., West C.M. (2017). O_2_ sensing-associated glycosylation exposes the F-box-combining site of the *Dictyostelium* Skp1 subunit in E3 ubiquitin ligases. J. Biol. Chem..

[36] Samuelson J., Banerjee S., Magnelli P., Cui J., Kelleher D.J., Gilmore R., Robbins P.W. (2005). The diversity of dolichol-linked precursors to Asn-linked glycans likely results from secondary loss of sets of glycosyltransferases. Proc. Natl. Acad. Sci. U. S. A..

[37] Banerjee S., Vishwanath P., Cui J., Kelleher D.J., Gilmore R., Robbins P.W., Samuelson J. (2007). The evolution of N-glycan-dependent endoplasmic reticulum quality control factors for glycoprotein folding and degradation. Proc. Natl. Acad. Sci. U. S. A..

[38] Paschinger K., Wilson I.B.H. (2019). Comparisons of N-glycans across invertebrate phyla. Version 2. Parasitology.

[39] Kurz S., Jin C., Hykollari A., Gregorich D., Giomarelli B., Vasta G.R., Wilson I.B.H., Paschinger K. (2013). Hemocytes and plasma of the eastern oyster (*Crassostrea virginica*) display a diverse repertoire of sulfated and blood group A-modified N-glycans. J. Biol. Chem..

[40] Zhou H., Hanneman A.J., Chasteen N.D., Reinhold V.N. (2013). Anomalous N-glycan structures with an internal fucose branched to GlcA and GlcN residues isolated from a mollusk shell-forming fluid. J. Proteome Res..

[41] Eckmair B., Jin C., Abed-Navandi D., Paschinger K. (2016). Multistep fractionation and mass spectrometry reveal zwitterionic and anionic modifications of the N- and O-glycans of a marine snail. Mol. Cell. Proteomics.

[42] Hykollari A., Malzl D., Eckmair B., Vanbeselaere J., Scheidl P., Jin C., Karlsson N.G., Wilson I.B.H., Paschinger K. (2018). Isomeric separation and recognition of anionic and zwitterionic N-glycans from royal jelly glycoproteins. Mol. Cell. Proteomics.

[43] Smit C.H., van Diepen A., Nguyen D.L., Wuhrer M., Hoffmann K.F., Deelder A.M., Hokke C.H. (2015). Glycomic analysis of life stages of the human parasite *Schistosoma mansoni* reveals developmental expression profiles of functional and antigenic glycan motifs. Mol. Cell. Proteomics.

[44] Vanbeselaere J., Jin C., Eckmair B., Wilson I.B.H., Paschinger K. (2020). Sulfated and sialylated N-glycans in the echinoderm *Holothuria atra* reflect its marine habitat and phylogeny. J. Biol. Chem..

[45] Cabrera G., Salazar V., Montesino R., Támbara Y., Struwe W.B., Leon E., Harvey D.J., Lesur A., Rincón M., Domon B., Méndez M., Portela M., González-Hernández A., Triguero A., Durán R. (2016). Structural characterization and biological implications of sulfated N-glycans in a serine protease from the neotropical moth *Hylesia metabus* (Cramer [1775]) (Lepidoptera: Saturniidae). Glycobiology.

[46] Martini F., Eckmair B., Štefanić S., Jin C., Garg M., Yan S., Jiménez-Castells C., Hykollari A., Neupert C., Venco L., Varón Silva D., Wilson I.B.H., Paschinger K. (2019). Highly modified and immunoactive N-glycans of the canine heartworm. Nat. Commun..

[47] Koles K., Lim J.-M., Aoki K., Porterfield M., Tiemeyer M., Wells L., Panin V. (2007). Identification of N-glycosylated proteins from the central nervous system of *Drosophila melanogaster*. Glycobiology.

[48] Geyer H., Wuhrer M., Resemann A., Geyer R. (2005). Identification and characterization of keyhole limpet hemocyanin N-glycans mediating cross-reactivity with *Schistosoma mansoni*. J. Biol. Chem..

[49] Yan S., Vanbeselaere J., Jin C., Blaukopf M., Wöls F., Wilson I.B.H., Paschinger K. (2018). Core richness of N-glycans of *Caenorhabditis elegans*: A case study on chemical and enzymatic release. Anal. Chem..

[50] Bushkin G.G., Ratner D.M., Cui J., Banerjee S., Duraisingh M.T., Jennings C.V., Dvorin J.D., Gubbels M.J., Robertson S.D., Steffen M., O'Keefe B.R., Robbins P.W., Samuelson J. (2010). Suggestive evidence for Darwinian selection against asparagine-linked glycans of *Plasmodium falciparum* and *Toxoplasma gondii*. Eukaryot. Cell.

[51] Schiller B., Hykollari A., Yan S., Paschinger K., Wilson I.B.H. (2012). Complicated N-linked glycans in simple organisms. Biol. Chem..

[52] Funk V.A., Thomas-Oates J.E., Kielland S.L., Bates P.A., Olafson R.W. (1997). A unique, terminally glucosylated oligosaccharide is a common feature on *Leishmania* cell surfaces. Mol. Biochem. Parasitol..

[53] Gas-Pascual E., Ichikawa H.T., Sheikh M.O., Serji M.I., Deng B., Mandalasi M., Bandini G., Samuelson J., Wells L., West C.M. (2019). CRISPR/Cas9 and glycomics tools for *Toxoplasma* glycobiology. J. Biol. Chem..

[54] Couto A.S., Gonçalves M.F., Colli W., de Lederkremer R.M. (1990). The N-linked carbohydrate chain of the 85-kilodalton glycoprotein from *Trypanosoma cruzi* trypomastigotes contains sialyl, fucosyl and galactosyl (α1-3)galactose units. Mol. Biochem. Parasitol..

[55] Barboza M., Duschak V.G., Fukuyama Y., Nonami H., Erra-Balsells R., Cazzul J.J., Couto A.S. (2005). Structural analysis of the N-glycans of the major cysteine proteinase of *Trypanosoma cruzi*. Identification of sulfated high-mannose type oligosaccharides. FEBS J..

[56] Couto A.S., Katzin A.M., Colli W., de Lederkremer R.M. (1987). Sialic acid in a complex oligosaccharide chain of the Tc-85 surface glycoprotein from the trypomastigote stage of *Trypanosoma cruzi*. Mol. Biochem. Parasitol..

[57] Damerow M., Graalfs F., Güther M.L., Mehlert A., Izquierdo L., Ferguson M.A. (2016). A gene of the β3-glycosyltransferase family encodes N-acetylglucosaminyltransferase II function in *Trypanosoma brucei*. J. Biol. Chem..

[58] Damerow M., Rodrigues J.A., Wu D., Güther M.L., Mehlert A., Ferguson M.A. (2014). Identification and functional characterization of a highly divergent N-acetylglucosaminyltransferase I (TbGnTI) in *Trypanosoma brucei*. J. Biol. Chem..

[59] Jinnelov A., Ali L., Tinti M., Güther M.S., Ferguson M.A.J. (2017). Single-subunit oligosaccharyltransferases of *Trypanosoma brucei* display different and predictable peptide acceptor specificities. J. Biol. Chem..

[60] Zielinska D.F., Gnad F., Schropp K., Wiśniewski J.R., Mann M. (2012). Mapping N-glycosylation sites across seven evolutionarily distant species reveals a divergent substrate proteome despite a common core machinery. Mol. Cell.

[61] Xu S.L., Medzihradszky K.F., Wang Z.Y., Burlingame A.L., Chalkley R.J. (2016). N-glycopeptide profiling in *Arabidopsis* inflorescence. Mol. Cell. Proteomics.

[62] Alves M.J.M., Kawahara R., Viner R., Colli W., Mattos E.C., Thaysen-Andersen M., Larsen M.R., Palmisano G. (2017). Comprehensive glycoprofiling of the epimastigote and trypomastigote stages of *Trypanosoma cruzi*. J. Proteomics.

[63] Luo Q., Upadhya R., Zhang H., Madrid-Aliste C., Nieves E., Kim K., Angeletti R.H., Weiss L.M. (2011). Analysis of the glycoproteome of *Toxoplasma gondii* using lectin affinity chromatography and tandem mass spectrometry. Microbes Infect..

[64] Feasley C.L., Johnson J.M., West C.M., Chia C.P. (2010). Glycopeptidome of a heavily N-glycosylated cell surface glycoprotein of *Dictyostelium* implicated in cell adhesion. J. Proteome Res..

[65] Mathieu-Rivet E., Scholz M., Arias C., Dardelle F., Schulze S., Le Mauff F., Teo G., Hochmal A.K., Blanco-Rivero A., Loutelier-Bourhis C., Kiefer-Meyer M.C., Fufezan C., Burel C., Lerouge P., Martinez F. (2013). Exploring the N-glycosylation pathway in *Chlamydomonas reinhardtii* unravels novel complex structures. Mol. Cell. Proteomics.

[66] West C.M., van der Wel H., Coutinho P.M., Henrissat B., Loomis W.F., Kuspa A. (2005). *Dictyostelium* Genomics.

[67] Sucgang R., Kuo A., Tian X., Salerno W., Parikh A., Feasley C.L., Dalin E., Tu H., Huang E., Barry K., Lindquist E., Shapiro H., Bruce D., Schmutz J., Salamov A. (2011). Comparative genomics of the social amoebae *Dictyostelium discoideum* and *Dictyostelium purpureum*. Genome Biol..

[68] O'Neill E.C., Kuhaudomlarp S., Rejzek M., Fangel J.U., Alagesan K., Kolarich D., Willats W.G.T., Field R.A. (2017). Exploring the glycans of *Euglena gracilis*. Biology (Basel).

[69] Joshi H.J., Narimatsu Y., Schjoldager K.T., Tytgat H.L.P., Aebi M., Clausen H., Halim A. (2018). SnapShot: O-glycosylation pathways across kingdoms. Cell.

[70] de Las Rivas M., Lira-Navarrete E., Gerken T.A., Hurtado-Guerrero R. (2019). Polypeptide GalNAc-Ts: From redundancy to specificity. Curr. Opin. Cell Biol..

[71] Roper J.R., Ferguson M.A. (2003). Cloning and characterisation of the UDP-glucose 4’-epimerase of *Trypanosoma cruzi*. Mol. Biochem. Parasitol..

[72] Wang F., Metcalf T., van der Wel H., West C.M. (2003). Initiation of mucin-type O-glycosylation in *Dictyostelium* is homologous to the corresponding step in animals and is important for spore coat function. J. Biol. Chem..

[73] Heise N., Singh D., van der Wel H., Sassi S.O., Johnson J.M., Feasley C.L., Koeller C.M., Previato J.O., Mendonça-Previato L., West C.M. (2009). Molecular analysis of a UDP-GlcNAc:polypeptide alpha-N-acetylglucosaminyltransferase implicated in the initiation of mucin-type O-glycosylation in *Trypanosoma cruzi*. Glycobiology.

[74] West C.M., van Der Wel H., Sassi S., Gaucher E.A. (2004). Cytoplasmic glycosylation of protein-hydroxyproline and its relationship to other glycosylation pathways. Biochim. Biophys. Acta.

[75] Stwora-Wojczyk M.M., Kissinger J.C., Spitalnik S.L., Wojczyk B.S. (2004). O-glycosylation in *Toxoplasma gondii*: Identification and analysis of a family of UDP-GalNAc:polypeptide N-acetylgalactosaminyltransferases. Int. J. Parasitol..

[76] DeCicco RePass M.A., Bhat N., Heimburg-Molinaro J., Bunnell S., Cummings R.D., Ward H.D. (2018). Molecular cloning, expression, and characterization of UDP N-acetyl-α-d-galactosamine: Polypeptide N-acetylgalactosaminyltransferase 4 from *Cryptosporidium parvum*. Mol. Biochem. Parasitol..

[77] Mendonça-Previato L., Penha L., Garcez T.C., Jones C., Previato J.O. (2013). Addition of α-O-GlcNAc to threonine residues define the post-translational modification of mucin-like molecules in *Trypanosoma cruzi*. Glycoconj. J..

[78] Riley G.R., West C.M., Henderson E.J. (1993). Cell differentiation in *Dictyostelium discoideum* controls assembly of protein-linked glycans. Glycobiology.

[79] Neubert P., Strahl S. (2016). Protein O-mannosylation in the early secretory pathway. Curr. Opin. Cell Biol..

[80] Sheikh M.O., Halmo S.M., Wells L. (2017). Recent advancements in understanding mammalian O-mannosylation. Glycobiology.

[81] Larsen I.S.B., Narimatsu Y., Clausen H., Joshi H.J., Halim A. (2019). Multiple distinct O-mannosylation pathways in eukaryotes. Curr. Opin. Struct. Biol..

[82] Mahne M., Tauch A., Puhler A., Kalinowski J. (2006). The *Corynebacterium glutamicum* gene pmt encoding a glycosyltransferase related to eukaryotic protein-O-mannosyltransferases is essential for glycosylation of the resuscitation promoting factor (Rpf2) and other secreted proteins. FEMS Microbiol. Lett..

[83] Holdener B.C., Haltiwanger R.S. (2019). Protein O-fucosylation: Structure and function. Curr. Opin. Struct. Biol..

[84] Takeuchi H., Schneider M., Williamson D.B., Ito A., Takeuchi M., Handford P.A., Haltiwanger R.S. (2018). Two novel protein O-glucosyltransferases that modify sites distinct from POGLUT1 and affect Notch trafficking and signaling. Proc. Natl. Acad. Sci. U. S. A..

[85] Li Z., Fischer M., Satkunarajah M., Zhou D., Withers S.G., Rini J.M. (2017). Structural basis of notch O-glucosylation and O-xylosylation by mammalian protein-O-glucosyltransferase 1 (POGLUT1). Nat. Commun..

[86] Yu H., Takeuchi H. (2019). Protein O-glucosylation: Another essential role of glucose in biology. Curr. Opin. Struct. Biol..

[87] Ogawa M., Sawaguchi S., Furukawa K., Okajima T. (2015). N-acetylglucosamine modification in the lumen of the endoplasmic reticulum. Biochim. Biophys. Acta.

[88] Bandini G., Albuquerque-Wendt A., Hegermann J., Samuelson J., Routier F.H. (2019). Protein O- and C-glycosylation pathways in *Toxoplasma gondii* and *Plasmodium falciparum*. Parasitology.

[89] Shcherbakova A., Preller M., Taft M.H., Pujols J., Ventura S., Tiemann B., Buettner F.F., Bakker H. (2019). C-mannosylation supports folding and enhances stability of thrombospondin repeats. eLife.

[90] Albuquerque-Wendt A., Hütte H.J., Buettner F.F.R., Routier F.H., Bakker H. (2019). Membrane topological model of glycosyltransferases of the GT-C superfamily. Int. J. Mol. Sci..

[91] Mreyen M., Champion A., Srinivasan S., Karuso P., Williams K.L., Packer N.H. (2000). Multiple O-glycoforms on the spore coat protein SP96 in *Dictyostelium discoideum*. Fuc(α1-3)GlcNAc-α-1-P-Ser is the major modification. J. Biol. Chem..

[92] Srikrishna G., Wang L., Freeze H.H. (1988). Fucoseβ-1-P-Ser is a new type of glycosylation: Using antibodies to identify a novel structure in *Dictyostelium discoideum* and study multiple types of fucosylation during growth and development. Glycobiology.

[93] Grabinska K.A., Ghosh S.K., Guan Z., Cui J., Raetz C.R.H., Robbins P.W., Samuelson J. (2008). Dolichyl-phosphate-glucose is used to make O-glycans on glycoproteins of *Trichomonas vaginalis*. Eukaryot. Cell.

[94] Pinger J., Nešić D., Ali L., Aresta-Branco F., Lilic M., Chowdhury S., Kim H.S., Verdi J., Raper J., Ferguson M.A.J., Papavasiliou F.N., Stebbins C.E. (2018). African trypanosomes evade immune clearance by O-glycosylation of the VSG surface coat. Nat. Microbiol..

[95] Showalter A.M., Basu D. (2016). Extensin and arabinogalactan-protein biosynthesis: Glycosyltransferases, research challenges, and biosensors. Front. Plant Sci..

[96] Canut H., Albenne C., Jamet E. (2016). Post-translational modifications of plant cell wall proteins and peptides: A survey from a proteomics point of view. Biochim. Biophys. Acta.

[97] Bollig K., Lamshöft M., Schweimer K., Marner F.J., Budzikiewicz H., Waffenschmidt S. (2007). Structural analysis of linear hydroxyproline-bound O-glycans of *Chlamydomonas reinhardtii*--conservation of the inner core in *Chlamydomonas* and land plants. Carbohydr. Res..

[98] Møller S.R., Yi X., Velásquez S.M., Gille S., Hansen P.L.M., Poulsen C.P., Olsen C.E., Rejzek M., Parsons H., Yang Z., Wandall H.H., Clausen H., Field R.A., Pauly M., Estevez J.M. (2017). Identification and evolution of a plant cell wall specific glycoprotein glycosyl transferase, ExAD. Sci. Rep..

[99] Hervé C., Siméon A., Jam M., Cassin A., Johnson K.L., Salmeán A.A., Willats W.G., Doblin M.S., Bacic A., Kloareg B. (2016). Arabinogalactan proteins have deep roots in eukaryotes: Identification of genes and epitopes in brown algae and their role in Fucus serratus embryo development. New Phytol..

[100] Spiro R.G., Lucas F., Rudall K.M. (1971). Glycosylation of hydroxylysine in collagens. Nat. New Biol..

[101] Moody-Haupt S., Patterson J.H., Mirelman D., McConville M.J. (2000). The major surface antigens of *Entamoeba histolytica* trophozoites are GPI-anchored proteophosphoglycans. J. Mol. Biol..

[102] Macrae J.I., Acosta-Serrano A., Morrice N.A., Mehlert A., Ferguson M.A. (2005). Structural characterization of NETNES, a novel glycoconjugate in *Trypanosoma cruzi* epimastigotes. J. Biol. Chem..

[103] Mule S.N., Saad J.S., Fernandes L.R., Stolf B.S., Cortez M., Palmisano G. (2020). Protein glycosylation in *Leishmania* spp. Mol. Omics.

[104] Reilly M.C., Aoki K., Wang Z.A., Skowyra M.L., Williams M., Tiemeyer M., Doering T.L. (2011). A xylosylphosphotransferase of *Cryptococcus neoformans* acts in protein O-glycan synthesis. J. Biol. Chem..

[105] Wang X.H., Nakayama K.-i., Shimma Y.-i., Tanaka A., Jigami Y. (1997). MNN6, a member of the KRE2/MNT1 family, is the gene for mannosylphosphate transfer in *Saccharomyces cerevisiae*. J. Biol. Chem..

[106] Qian Y., West C.M., Kornfeld S. (2010). UDP-GlcNAc:Glycoprotein N-acetylglucosamine-1-phosphotransferase mediates the initial step in the formation of the methylphosphomannosyl residues on the high mannose oligosaccharides of *Dictyostelium discoideum* glycoproteins. Biochem. Biophys. Res. Commun..

[107] Ivanova I.M., Nepogodiev S.A., Saalbach G., O'Neill E.C., Urbaniak M.D., Ferguson M.A., Gurcha S.S., Besra G.S., Field R.A. (2017). Fluorescent mannosides serve as acceptor substrates for glycosyltransferase and sugar-1-phosphate transferase activities in *Euglena gracilis* membranes. Carbohydr. Res..

[108] Chalkley R.J., Thalhammer A., Schoepfer R., Burlingame A.L. (2009). Identification of protein O-GlcNAcylation sites using electron transfer dissociation mass spectrometry on native peptides. Proc. Natl. Acad. Sci. U. S. A..

[109] Shou-Ling Xu S.L., Chalkley R.J., Maynard J.C., Wang W., Ni W., Jiang X., Shin K., Cheng L., Savage D., Hühmer A.F.R., Burlingame A.L., Wang Z.Y. (2017). Proteomic analysis reveals O-GlcNAc modification on proteins with key regulatory functions in *Arabidopsis*. Proc. Natl. Acad. Sci. U. S. A..

[110] Olszewski N.E., West C.M., Sassi S.O., Hartweck L.M. (2010). O-GlcNAc protein modification in plants: Evolution and function. Biochim. Biophys. Acta.

[111] Bandini G., Haserick J.R., Motari E., Ouologuem D.T., Lourido S., Roos D.S., Costello C.E., Robbins P.W., Samuelson J. (2016). O-fucosylated glycoproteins form assemblies in close proximity to the nuclear pore complexes of *Toxoplasma gondii*. Proc. Natl. Acad. Sci. U. S. A..

[112] Bandini G., Agop-Nersesian C., van der Wel H., Mandalasi M., Kim H.W., West C.M., Samuelson J. (2021). The nucleocytosolic O-fucosyltransferase Spindly affects protein expression and virulence in *Toxoplasma gondii*. J. Biol. Chem..

[113] Zentella R., Sui N., Barnhill B., Hsieh W.P., Hu J., Shabanowitz J., Boyce M., Olszewski N.E., Zhou P., Hunt D.F., Sun T.P. (2017). The *Arabidopsis* O-fucosyltransferase SPINDLY activates nuclear growth repressor DELLA. Nat. Chem. Biol..

[114] Curtino J.A., Aon M.A. (2019). From the seminal discovery of proteoglycogen and glycogenin to emerging knowledge and research on glycogen biology. Biochem. J..

[115] Halim A., Larsen I.S., Neubert P., Joshi H.J., Petersen B.L., Vakhrushev S.Y., Strahl S., Clausen H. (2015). Discovery of a nucleocytoplasmic O-mannose glycoproteome in yeast. Proc. Natl. Acad. Sci. U. S. A..

[116] Sheikh M.O., Xu Y., van der Wel H., Walden P., Hartson S.D., West C.M. (2015). Glycosylation of Skp1 promotes formation of Skp1-cullin-1-F-box protein complexes in *Dictyostelium*. Mol. Cell. Proteomics.

[117] Xu X., Eletsky A., Sheikh M.O., Prestegard J.H., West C.M. (2018). Glycosylation promotes the random coil to helix transition in a region of a protist Skp1 associated with F-box binding. Biochemistry.

[118] Sheikh M.O., Schafer C.M., Powell J.T., Rodgers K.K., Mooers B.H., West C.M. (2014). Glycosylation of Skp1 affects its conformation and promotes binding to a model F-box protein. Biochemistry.

[119] Shajahan A., Supekar N.T., Heiss C., Ishihara M., Azadi P. (2017). Tool for rapid analysis of glycopeptide by permethylation via one-pot site mapping and glycan analysis. Anal. Chem..

[120] Mandalasi M., Kim H.W., Thieker D., Sheikh M.O., Gas-Pascual E., Rahman K., Zhao P., Daniel N.G., van der Wel H., Ichikawa H.T., Glushka J.N., Wells L., Woods R.J., Wood Z.A., West C.M. (2020). A terminal α3-galactose modification regulates an E3 ubiquitin ligase subunit in *Toxoplasma gondii*. J. Biol. Chem..

[121] van der Wel H., Gas-Pascual E., West C.M. (2019). Skp1 isoforms are differentially modified by a dual function prolyl 4-hydroxylase/N-acetylglucosaminyltransferase in a plant pathogen. Glycobiology.

[122] Rahman K., Mandalasi M., Zhao P., Sheikh M.O., Taujale R., Kim H.W., van der Wel H., Matta K., Kannan N., Glushka J.N., Wells L., West C.M. (2017). Characterization of a cytoplasmic glucosyltransferase that extends the core trisaccharide of the *Toxoplasma* Skp1 E3 ubiquitin ligase subunit. J. Biol. Chem..

[123] Schafer C.M., Sheikh M.O., Zhang D., West C.M. (2014). Novel regulation of Skp1 by the *Dictyostelium* AgtA α-galactosyltransferase involves the Skp1-binding activity of its WD40 repeat domain. J. Biol. Chem..

[124] van der Wel H., Fisher S.Z., West C.M. (2002). A bifunctional diglycosyltransferase forms the Fucα1,2Galβ1,3-disaccharide on Skp1 in the cytoplasm of *Dictyostelium*. J. Biol. Chem..

[125] van Der Wel H., Morris H.R., Panico M., Paxton T., Dell A., Kaplan L., West C.M. (2002). Molecular cloning and expression of a UDP-N-acetylglucosamine (GlcNAc):hydroxyproline polypeptide GlcNAc-transferase that modifies Skp1 in the cytoplasm of *Dictyostelium*. J. Biol. Chem..

[126] West C.M., Blader I.J. (2015). Oxygen sensing by protozoans: How they catch their breath. Curr. Opin. Microbiol..

